# Genetic relationships between suicide attempts, suicidal ideation and major psychiatric disorders: A genome-wide association and polygenic scoring study

**DOI:** 10.1002/ajmg.b.32247

**Published:** 2014-06-25

**Authors:** Niamh Mullins, Nader Perroud, Rudolf Uher, Amy W Butler, Sarah Cohen-Woods, Margarita Rivera, Karim Malki, Jack Euesden, Robert A Power, Katherine E Tansey, Lisa Jones, Ian Jones, Nick Craddock, Michael J Owen, Ania Korszun, Michael Gill, Ole Mors, Martin Preisig, Wolfgang Maier, Marcella Rietschel, John P Rice, Bertram Müller-Myhsok, Elisabeth B Binder, Susanne Lucae, Marcus Ising, Ian W Craig, Anne E Farmer, Peter McGuffin, Gerome Breen, Cathryn M Lewis

**Affiliations:** 1 MRC Social, Genetic and Developmental Psychiatry Centre, Institute of Psychiatry, King's College LondonLondon, United Kingdom; 2 Psychiatry, University Hospital of GenevaGeneva, Switzerland; 3 Department of Psychiatry, Dalhousie UniversityHalifax, Nova Scotia, Canada; 4 Department of Psychiatry, University of Hong KongHong Kong, Special Administrative Region, China; 5 Department of Psychiatry, University of AdelaideAdelaide, Australia; 6 Centro de Investigación Biomédica en Red de Salud Mental, CIBERSAM, University of GranadaGranada, Spain; 7 MRC Centre for Neuropsychiatric Genetics and Genomics, Neuroscience and Mental Health Research Institute, Cardiff UniversityCardiff, United Kingdom; 8 Department of Psychiatry, School of Clinical and Experimental Medicine, University of BirminghamBirmingham, United Kingdom; 9 Barts and The London Medical School, Queen Mary University of LondonLondon, United Kingdom; 10 Department of Psychiatry, Trinity Centre for Health ScienceDublin, Ireland; 11 Research Department P, Aarhus University HospitalRisskov, Denmark; 12 University Hospital Center and University of LausanneLausanne, Switzerland; 13 Department of Psychiatry, University of BonnBonn, Germany; 14 Division of Genetic Epidemiology in Psychiatry, Central Institute of Mental HealthMannheim, Germany; 15 Department of Psychiatry, Washington University, St. LouisMissouri; 16 Max Planck Institute of PsychiatryMunich, Germany; 17 NIHR Biomedical Research Centre for Mental Health, South London and Maudsley NHS Foundation Trust and Institute of Psychiatry, King's College LondonLondon, United Kingdom; 18 Division of Genetics and Molecular Medicine, King's College London School of Medicine, Guy's HospitalLondon, United Kingdom

**Keywords:** pleiotropy, meta-analysis, prediction, association

## Abstract

Epidemiological studies have recognized a genetic diathesis for suicidal behavior, which is independent of other psychiatric disorders. Genome-wide association studies (GWAS) on suicide attempt (SA) and ideation have failed to identify specific genetic variants. Here, we conduct further GWAS and for the first time, use polygenic score analysis in cohorts of patients with mood disorders, to test for common genetic variants for mood disorders and suicide phenotypes. Genome-wide studies for SA were conducted in the RADIANT and GSK-Munich recurrent depression samples and London Bipolar Affective Disorder Case-Control Study (BACCs) then meta-analysis was performed. A GWAS on suicidal ideation during antidepressant treatment had previously been conducted in the Genome Based Therapeutic Drugs for Depression (GENDEP) study. We derived polygenic scores from each sample and tested their ability to predict SA in the mood disorder cohorts or ideation status in the GENDEP study. Polygenic scores for major depressive disorder, bipolar disorder and schizophrenia from the Psychiatric Genomics Consortium were used to investigate pleiotropy between psychiatric disorders and suicide phenotypes. No significant evidence for association was detected at any SNP in GWAS or meta-analysis. Polygenic scores for major depressive disorder significantly predicted suicidal ideation in the GENDEP pharmacogenetics study and also predicted SA in a combined validation dataset. Polygenic scores for SA showed no predictive ability for suicidal ideation. Polygenic score analysis suggests pleiotropy between psychiatric disorders and suicidal ideation whereas the tendency to *act* on such thoughts may have a partially independent genetic diathesis. © 2014 The Authors. American Journal of Medical Genetics Part B: Neuropsychiatric Genetics published by Wiley Periodicals, Inc.

## Introduction

Suicide is one of the ten leading causes of death and rates of suicide are far exceeded by suicide attempts, which occur up to 20 times more frequently [World Health Organisation, 2005; Lim et al., 2012]. Over 90% of suicide attempters or victims have a psychiatric diagnosis, particularly major depression and bipolar disorder [Beautrais et al., 1996; Qin, 2011]. However, twin, family and adoption studies have recognized a genetic component to suicidal behavior, which is independent of other psychiatric disorders [Schulsinger et al., 1979; Brent et al., 1996; Roy and Segal, 2001]. This supports a stress-diathesis model whereby psychiatric illnesses or other life stressors are not exclusively responsible for suicide attempts (SA) but rather that they may *aggravate* an individual's genetic predisposition to suicidal behavior [Mann et al., 1999].

Candidate gene association studies on suicidal behavior have yielded either inconsistent or negative results. Similarly, genome-wide association studies (GWAS) performed to date have failed to identify associations at the genome-wide significance level [Perlis et al., 2010; Schosser et al., 2011; Willour et al., 2012; Galfalvy et al., 2013]. Other genetic studies have focused on treatment emergent or treatment worsening suicidal ideation (SI) in patients taking antidepressant drugs [Laje et al., 2009; Perroud et al., 2012].

One model for suicidal behavior is as a complex trait, which occurs when the joint effect of many risk and protective genetic variants, together with an environmental contribution, crosses a certain liability threshold. GWAS captures alleles that are common in the population and are each likely to have a very small effect on a complex phenotype [Wray et al., 2007]. Simultaneously investigating thousands of SNPs, which individually do not reach statistical significance, could explain a greater amount of the heritability. Polygenic score analysis uses ensembles of SNPs from a GWAS in a discovery sample, weighted by their log odds ratios to create a score across all these alleles for each individual in an independent validation sample [Dudbridge, 2013]. The polygenic score can then be tested for its ability to predict case and control status in the validation dataset or to predict a different phenotype in the validation dataset, to investigate pleiotropy between traits [Purcell et al., 2009].

Suicidal behavior and ideation have been associated with most psychiatric and behavioral disorders. Pleiotropic genetic effects have been identified between several disorders which have shared symptoms or are commonly comorbid, using polygenic scores for one disorder to predict another [Purcell et al., 2009].

GWAS on suicide attempt in patients with mood disorders may detect genetic variants predisposing to SA which are independent of psychiatric disorders and a meta-analysis may identify novel associations and reduce false positive results. Polygenic score analysis could explain a larger component of the heritability of suicidal behavior, than would individual alleles with small effect sizes. There may be pleiotropic genetic effects between suicide attempt or ideation and depression, schizophrenia or bipolar disorder, since suicidality is a common comorbidity in these illnesses.

In this article, we test for genetic associations with suicide attempt and suicidal ideation in four studies of depression and bipolar disorder. Using these, and results from the Psychiatric Genomics Consortium, we construct polygenic scores and test their predictive ability for suicide attempt and ideation.

## Materials and Methods

### Clinical Sample Collection

A GWAS on SA has previously been conducted in the RADIANT sample (n = 2,023) of patients with recurrent major depressive disorder [Schosser et al., 2011]. Briefly, patients were diagnosed using the Schedules for Clinical Assessment in Neuropsychiatry Interview (SCAN), according to standardised criteria in the International Classification of Diseases 10th edition (ICD-10) or Diagnostic and Statistical Manual 4th edition (DSM-IV) [World Health Organisation, 1998; Wing et al., 1990; American Psychiatric Association, 2000]. The SCAN was used to record information on patients' worst and second worst episodes of depression including suicidal behavior. Exclusion criteria have been described previously and include Axis I diagnoses other than anxiety disorder [Farmer et al., 2004; Cohen-Woods et al., 2009].

The GSK-Munich recurrent depression cohort (n = 807) was recruited at the Max-Planck Institute of Psychiatry, Germany according to the same protocol [Muglia et al., 2010].

Participants in the Bipolar Affective Disorder Case Control Study (BACCs) (n = 470) were recruited in London, United Kingdom in the same manner and assessed for diagnosis using the SCAN interview and Operational Criteria Checklist for Psychotic Illness Program [McGuffin et al., 1991]. Exclusion criteria have been described previously [Gaysina et al., 2009].

A GWAS on treatment-emergent and treatment-worsening suicidal ideation has also been reported in the Genome Based Therapeutic Drugs for Depression Study (GENDEP), comprised of patients with major depression on a 12 week antidepressant treatment with either escitalopram—a selective serotonin reuptake inhibiter or nortriptyline—a tricylic antidepressant [Uher et al., 2010; Perroud et al., 2012].

Participants in all studies were of European ancestry. Whole blood samples were collected in ethylene-diamine-tetra-acetic acid (EDTA) for genetic analysis.

### Phenotype Definitions

Suicide attempt was defined using a question on the SCAN interview, as self-injury or suicide attempt resulting in serious harm, or an attempt at suicide designed to result in death [Schosser et al., 2011]. Evidence collected retrospectively for the worst and second worst depressive episode was used in this study.

In the GENDEP study, participants were evaluated weekly using the 17-item Hamilton Rating Scale for Depression, the self-report Beck Depression Inventory and the Montgomery-Asberg Depression Rating Scale, which each include a question on suicidal ideation [Hamilton, 1960; Beck et al., 1961; Montgomery and Asberg, 1979]. A composite score was used to define suicidal ideation at baseline, treatment emergent (TESI) and treatment worsening suicidal ideation (TWOSI) [Perroud et al., 2009]. Individuals with either TWOSI or TESI were considered as cases and those with no suicidal ideation or baseline ideation which did not worsen during treatment were used as controls.

### Genotyping

DNA was extracted from blood samples and those of sufficient quantity and quality were genotyped [Freeman et al., 2003]. GSK-Munich and BACCs samples were genotyped on Illumina HumanHap550-Quad+ BeadChip microarrays (Illumina, Inc., San Diego, CA). RADIANT and GENDEP samples were genotyped on Illumina Human610-Quad BeadChip (Illumina, Inc.) by the Centre National de Génotypage (Evry Cedex, France).

### Statistical Analysis

Standard quality control procedures were implemented in all studies to clean the data and control for population stratification [Lewis et al., 2010; Schosser et al., 2011; Perroud et al., 2012].

GWAS on SA were performed by logistic regression using the whole genome analysis toolset PLINK 1.07 (http://pngu.mgh.harvard.edu/purcell/plink/) [Purcell et al., 2007] in the GSK-Munich and BACCs samples and have previously been reported in RADIANT [Schosser et al., 2011]. A GWAS on TESI/TWOSI has also been reported in GENDEP [Perroud et al., 2012]. To account for multiple testing, genome wide significance was ascribed at P < 5 × 10^−8^ and findings of suggestive significance at P < 5 × 10^−6^ [Dudbridge and Gusnanto, 2008]. Principal components were included as covariates to account for population stratification and the median chi-squared test statistic (*λ*) was used to test the quality of control [Devlin et al., 2001]. This was close to 1 in all studies (Supplementary material), suggesting no inflation of test statistics and so the unadjusted *P* values were reported.

A fixed effects meta-analysis on SA was performed between the RADIANT, GSK-Munich, and BACCs studies, using inverse variance weighting to combine the results [Evangelou and Ioannidis, 2013].

Assuming a discrete trait Case–Control model, the Genetic Power Calculator [Purcell et al., 2003] was used to determine the power to detect association at both significance levels, for the individual studies and meta-analysis (Supplementary material).

The GWAS results from these four discovery studies were pruned for linkage disequilibrium (LD) using the *P*-value informed clumping method in PLINK. This preferentially retains SNPs with the strongest evidence of association and removes SNPs in LD that show weaker evidence of association. Subsets of SNPs were selected from the results at 5 increasingly liberal P value thresholds (*P* < 0.01, *P* < 0.05, *P* < 0.1, *P* < 0.3, *P* < 0.5). Sets of alleles, weighted by their original log odds ratios (OR) in the GWAS, were summarized into a polygenic score for each individual in the different validation datasets using PLINK. The polygenic score was tested for ability to predict SA or TWOSI/TESI in the validation sample using logistic regression, with principal components as required, to calculate Nagelkerke's pseudo-R^2^, a measure of the variance explained [Nagelkerke, 1991].

The same procedures were followed using GWAS summary results from the Psychiatric Genomics Consortium available online (https://pgc.unc.edu/). Polygenic scores for major depressive disorder (PGC-MDD), bipolar disorder (PGC-BIP) and schizophrenia (PGC-SCZ) were tested for ability to predict SA or TWOSI/TESI in each of the four validation samples [Psychiatric GWAS Consortium Bipolar Disorder Working Group, 2011; Schizophrenia Psychiatric Genome-Wide Association Study (GWAS) Consortium, 2011]. In the case of PGC-MDD, published results were from a mega-analysis of nine studies, which included the RADIANT and GSK-Munich samples [Major Depressive Disorder Working Group of the Psychiatric Genomics Consortium, 2013]. Meta-analyses were repeated using summary results from the individual studies and excluding the RADIANT or GSK-Munich sample as required, avoiding overlap between discovery and validation datasets, which could cause false positive results. Finally, the RADIANT, GSK-Munich and BACCs datasets were also combined into one larger validation sample and polygenic scores for psychiatric disorders from the PGC studies in MDD, SCZ, and BIP were tested as predictors of SA. Statistical analysis was performed using R 3.0.0.

A literature review was performed using Pubmed http://www.ncbi.nlm.nih.gov/pubmed/ to investigate genes previously associated with suicidal ideation, suicide attempt or related endophenotypes for a candidate association study. All genotyped SNPs within the selected candidate genes were extracted from the meta-analysis results. SNPs were pruned for LD by *P* value informed clumping and the threshold for significance was calculated using a Bonferroni correction for the number of SNPs tested (Supplementary material).

### Ethical Approval

All patients gave written informed consent to take part in these studies. Data were anonymized with a research identification number only. Ethical approval was obtained from the Ethics Committee at the Institute of Psychiatry, King's College London or the local ethics committee at each center of recruitment.

## Results

### Sample Characteristics

Sample characteristics are shown in TableI. A similar proportion of cases in RADIANT, GSK-Munich, and BACCs studies were suicide attempters (12–16%), while rates of TESI/TWOSI in the GENDEP study were higher (32%).

**Table I:** Sample Characteristics

Sample	No. individuals	No. SA or TESI/TWOSI cases (%)	No. females (%)	Mean age onset, SD (years)	Mean age at interview, SD (years)
RADIANT^a^	2,023	251 (12.4%)	1,477 (73.0%)	22.7 (11.5)	46.2 (12.0)
GSK-Munich^a^	807	106 (13.1%)	531 (65.8%)	35.8 (14.1)	51.1 (13.8)
BACCs^a^	440	69 (15.7%)	294 (66.8%)	21.7 (8.4)	47.7 (11.5)
Meta-analysis^b^	3,270	426 (13.0%)	2,302 (70.4%)	26.0 (13.3)	47.6 (12.5)
GENDEP^c^	747	237 (31.7%)	470 (62.9%)	32.1 (10.4)	41.9 (11.7)

a Cases are suicide attempters.

b RADIANT, GSK-Munich, and BACCs studies.

c Cases in the GENDEP study are those with suicidal ideation.

### Genome-Wide Association Analysis

No SNP reached genome-wide significance in the GWAS on SA in GSK-Munich and BACCs (Supplementary material). rs9351947 on chromosome 6 reached suggestive significance in the GSK-Munich analysis, whereas the BACCs sample was underpowered to detect such effects (power calculations shown in Supplementary material).

**Figure 1:**
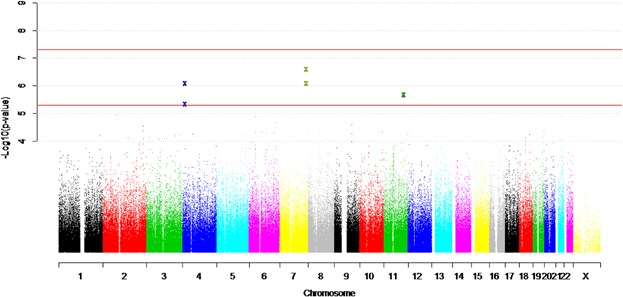
Manhattan plot of meta-analysis between RADIANT, GSK-Munich, and BACCs samples.

The fixed effects meta-analysis performed between the RADIANT, GSK-Munich and BACCs studies on SA, had much higher power to detect association (87% power at the suggestive significance level for a minor allele with a frequency of 0.2 and OR = 1.5 and 57% power at genome-wide significance). No SNP reached genome-wide significance (TableII). SNPs on chromosomes 7, 4 and 11 reached suggestive significance, and had not reached this threshold in the individual study analysis (Fig. 1). Meta-analysis did not provide any further support for any of the SNPs of suggestive significance in the individual studies (Supplementary material).

**Table II:** Fixed Effects Meta-Analysis of RADIANT, GSK-Munich, and BACCs results in an Additive Genetic Model Showing the Most Significant SNP From Each Genomic Region

SNP	CHR	BP	Tested allele	Allele freq^a^	Closest gene^b^	Meta-analysis		I^2^ (%)	RADIANT		GSK-Munich		BACCs	
						*P* value	OR (C.I.)		*P* value	OR	*P* value	OR	*P* value	OR
rs17173608	7	149667597	G	0.058	*RARRES2*	2.41E−07	1.93 (1.69–2.19)	0	1.52E−04	1.92	1.59E−03	2.16	9.57E−02	1.67
rs17387100	4	15604223	G	0.078	*PROM1*	7.98E−07	1.76 (1.54–1.99)	8.4	1.00E−05	1.92	3.59E−01	1.26	1.28E−02	1.92
rs3781878	11	112625422	A	0.272	*NCAM1*	1.98E−06	0.65 (0.48–0.83)	0	2.11E−03	0.70	6.61E−03	0.61	8.83E−03	0.56
rs17010519	2	74995810	C	0.493	*HK2*	1.15E−05	1.39 (1.24–1.54)	0	5.05E−04	1.41	2.41E−02	1.40	1.42E−01	1.32
rs13049531	21	34916581	A	0.086	*RCAN1*	1.27E−05	1.65 (1.43–1.88)	0	2.44E−03	1.56	2.31E−02	1.75	2.41E−02	1.86
rs9394433	6	37488302	C	0.400	*RNF8*	1.30E−05	1.38 (1.24–1.54)	0	3.17E−04	1.42	2.36E−01	1.20	1.31E−02	1.61
rs10089628	8	13440033	T	0.366	*DLC1*	1.89E−05	0.70 (0.55–0.87)	0	8.85E−05	0.66	9.55E−02	0.77	2.54E−01	0.80
rs12173791	6	151866142	A	0.082	*CCDC170*	2.12E−05	1.66 (1.44–1.90)	0	3.06E−04	1.75	2.85E−02	1.72	3.40E−01	1.33
rs7035325	9	96449258	A	0.082	*FBP1*	2.55E−05	1.67 (1.43–1.91)	0	9.35E−04	1.66	3.95E−02	1.74	1.10E−01	1.67
rs2030199	2	225969851	G	0.174	*NYAP2*	2.82E−05	0.62 (0.41–0.85)	0	8.47E−04	0.62	3.72E−02	0.61	1.39E−01	0.68

CHR, chromosome; BP, basepair position; OR, odds ratio; CI, confidence interval; I^2^, heterogeneity index.

a Allele frequencies are from the RADIANT study.

b The closest gene to each SNP is shown, as determined using Ensembl.

### Candidate Association Study

From the meta-analysis, a candidate association study of 19 genes previously associated with suicide was also performed though no SNPs were significant after a Bonferroni correction was applied (Supplementary material). A SNP in *ABI3BP* had reached GWAS significance in a previous study of SA in depression, however the finding did not replicate in this analysis [Perlis et al., 2010].

### Polygenic Score Analysis

**Figure 2:**
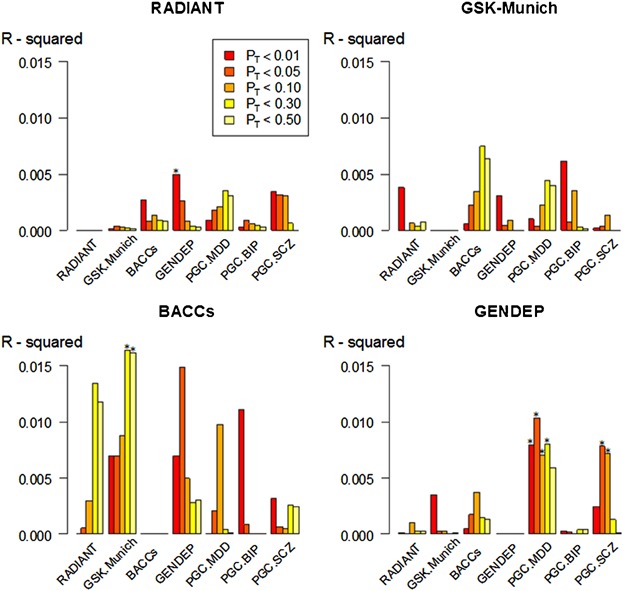
Polygenic scores for suicide attempt, suicidal ideation, and psychiatric disorders from seven discovery datasets used to predict suicide attempt in the RADIANT, GSK-Munich, and BACCs validation datasets and suicidal ideation in GENDEP. The y-axis indicates Nagelkerke's pseudo R^2^, a measure of the variance explained and the x-axis indicates the discovery datasets from which the polygenic scores were derived. The five *P*-value thresholds used (*P*_T_ < 0.01, *P*_T_ < 0.05, *P*_T_ < 0.10, *P*_T_ < 0.30, *P*_T_ < 0.50) to select SNPs are plotted left to right for each discovery dataset. Stars indicate polygenic scores with significant predictive ability (*P* < 0.05).

SNPs from four discovery GWAS on suicide attempt (RADIANT, GSK-Munich, BACCs), one study on suicidal ideation (GENDEP) and three studies on psychiatric disorders (PGC-MDD, PGC-BIP, PGC-SCZ) were used to derive polygenic scores for each individual in four validation datasets (RADIANT, GSK-Munich, BACCs, GENDEP). Subsets of SNPs were selected according to increasingly liberal *P* value thresholds (P_T_) in the discovery phase GWAS analysis. Scores were tested for their ability to predict suicide attempt or ideation in the validation samples by calculating Nagelkerke's pseudo R^2^, a measure of the variance explained (Fig. 2).

In the RADIANT validation sample, only polygenic scores for TESI/TWOSI derived from GENDEP showed significant predictive ability for suicide attempt (*P* = 0.021) (Fig. 2). The Nagelkerke R^2^ was 0.005 indicating that 0.5% of variance in the phenotype could be explained by SNPs within *P*_T_ < 0.01 of the GENDEP polygenic score, with no significant prediction obtained at other *P* value thresholds.

In the GSK-Munich validation dataset, none of the polygenic scores showed any significant predictive ability (Fig. 2). The *P*_T_ < 0.3 and *P*_T_ < 0.5 scores for SA from the GSK-Munich sample had predictive ability for SA in BACCs (R^2^ = 1.6%, *P* = 0.04).

Polygenic scores for major depressive disorder from the PGC were significant predictors of TESI/TWOSI in the GENDEP validation dataset (Fig. 2), accounting for 1% of TESI/TWOSI variance (*P*_T_ < 0.3, *P* = 0.015). Scores for schizophrenia also showed significant predictive ability in GENDEP, but were lower in cases experiencing TESI/TWOSI than controls with no ideation. Polygenic scores for SA showed no predictive ability for suicidal ideation during antidepressant treatment in GENDEP (Fig. 2).

The RADIANT, GSK-Munich, and BACCs validation datasets were merged into a single dataset in order to increase the precision of prediction. This sample has the same characteristics as the meta-analysis (n = 3,270) and includes 426 SA cases. Scores for MDD, BIP and SCZ from the PGC were tested as predictors of SA in this combined validation dataset (Supplementary material). PGC-MDD scores showed significant predictive ability for SA, the largest R^2^ being 0.3% at *P*_T_ < 0.3 (*P* = 0.013). Scores from PGC-SCZ also showed significant prediction (R^2^ = 0.2% at *P*_T_ < 0.05, *P* = 0.05), but the score was lower in SA cases than in non-SA cases, consistent with the previous analysis in the GENDEP sample.

## Discussion

Meta-analysis of the RADIANT, GSK-Munich, and BACCs studies found no genome-wide significant SNPs associated with SA (TableII), in common with most previous genome-wide association studies on suicide attempt. This probably reflects the limited power of our sample to detect the genetic contribution to a complex trait like SA in patients with mood disorders. A similar challenge is seen in detecting genetic associations with the underlying mood disorders, where the PGC-MDD study detected no significant loci despite using a sample of over 9,000 cases and 9,000 controls [Major Depressive Disorder Working Group of the Psychiatric Genomics Consortium, 2013].

The GENDEP study was excluded from the meta-analysis because of the different phenotype assessed (suicidal ideation, not suicide attempt), and its design (a prospective study during a 12-week treatment period rather than a retrospective study). There is considerable evidence in the literature that suicidal ideation is not part of the same phenotypic spectrum as SA. An excess of suicidal ideation was found in the relatives of suicide victims but was not significant after adjustment for psychiatric disorders [Brent et al., 1996]. It has been proposed that ideation co-segregates with psychiatric disorders, whereas the tendency to act upon such thoughts has a partially separate genetic diathesis [Mann et al., 1999]. This suggests that suicidal ideation should be excluded from molecular genetic studies on SA.

A SNP in *RARRES2* was the most significant finding in the meta-analysis (TableII). This gene encodes a retinoic acid receptor responder. Retinoic acid is a signaling molecule involved in neurogenesis and has been linked to depression and suicide [O'Reilly et al., 2006; Siegenthaler et al., 2009]. Previous GWAS and linkage studies on SA in different psychiatric disorders and meta-analysis between them, have implicated the 2p12 region [Hesselbrock et al., 2004; Willour et al., 2007; Willour et al., 2012; Butler et al., 2010]. Further support is obtained in this meta-analysis from rs17010519 in the *HK2* gene (*P* = 1.15 × 10^−5^) which is within the linkage region previously identified.

Although genome-wide association studies have low power to detect association at a single SNP in psychiatric phenotypes, polygenic score analysis provides an alternative approach, simultaneously testing ensembles of markers, which may not reach significance individually. This approach has been used by the Psychiatric Genomics Consortium and the International Schizophrenia Consortium to investigate major depression and schizophrenia [Purcell et al., 2009; Major Depressive Disorder Working Group of the Psychiatric Genomics Consortium, 2013].

Polygenic scores for SA (RADIANT, GSK-Munich, and BACCs), suicidal ideation (GENDEP) and depression, bipolar disorder and schizophrenia from the PGC, were tested for their ability to predict suicide attempt or ideation in four validation datasets. Some significant predictive ability was found using GENDEP scores for suicidal ideation to predict SA in the RADIANT validation dataset (Fig. 2). The highest R^2^ in our analysis was 1.6% using scores for SA from GSK-Munich to predict SA in the BACCs sample (Fig. 2), which is modest but is in line with previous results using polygenic scores for depression and schizophrenia, as predictors in independent samples, 0.6% and 3% respectively [Purcell et al., 2009; Major Depressive Disorder Working Group of the Psychiatric Genomics Consortium, 2013].

This is the first study to use polygenic score analysis on studies of suicide attempt and suicidal ideation, although lack of power is still a limitation, given the moderate sample size of most of the discovery data sets (RADIANT, GSK-Munich, BACCs, and GENDEP) [Dudbridge, 2013]. The size of the validation dataset determines the precision with which the phenotype can be predicted [Dudbridge, 2013]. Validation datasets used here may be too small and this could explain why the majority of polygenic scores did not yield significant *P* values. Suicide attempt has a heritability of 55%, so if the polygenic score explained half of the heritability of liability, a sample of many thousands of individuals would still be required to achieve a predictive ability of 75%, which is the standard for screening at risk populations [Voracek and Loibl, 2007; Dudbridge, 2013].

Polygenic scores for MDD, BIP and SCZ from the Psychiatric Genomics Consortium were tested for prediction of SA and suicidal ideation, to investigate possible pleiotropy between these commonly comorbid conditions.

Polygenic scores for psychiatric disorders showed no significant prediction of SA in any of the validation datasets individually (Fig. 2), but scores for MDD did predict SA in the RADIANT, GSK-Munich, and BACCs validation datasets combined (Supplementary material). The limitation of sample size in the discovery phase also applies to the MDD study by the PGC and may reduce the accuracy of this polygenic score [Major Depressive Disorder Working Group of the Psychiatric Genomics Consortium, 2013]. However, combining the validation datasets appears to have allowed more precise prediction which reached statistical significance. Scores for bipolar disorder showed no predictive ability in the combined validation sample, which may be due to the small number of patients with BIP included.

There has been much debate surrounding the relationship between psychiatric disorders and suicide attempt. Many epidemiological studies have shown that suicide and suicide attempt aggregates in families even after controlling for the presence of psychiatric disorders [Brent et al., 1996; Johnson et al., 1998]. Two pedigrees were reported with equal loading for mood disorder, one of which was also loaded for suicide while the other had not a single case of suicide [Egeland and Sussex, 1985]. It has been suggested that the familial transmission of suicide attempt is conditional on but independent of mood disorders [Turecki, 2001]. The results presented here are consistent with a partial pleiotropy between major depression and SA.

Polygenic scores for major depressive disorder significantly predicted suicidal ideation in the GENDEP pharmacogenetic study, suggesting a pleiotropy between liability to depression and TESI/TWOSI (Fig. 2). In contrast, polygenic scores reflecting liability to bipolar disorder did not predict TESI/TWOSI during antidepressant treatment (Fig. 2). These findings suggest that emergence or worsening of suicidal thoughts during treatment with antidepressants is a phenomenon inherent to the genetic liability to depression and is not a reflection of latent or misdiagnosed bipolar disorder [Tansey et al., 2014], as was previously suggested [Berk and Dodd, 2005; Seemuller et al., 2009]. Scores for suicide attempt did not predict TESI/TWOSI in GENDEP, further emphasizing the distinct genetic aetiology of suicidal ideation and suicidal behavior. It is important to note that TESI/TWOSI occurs in a minority of patients on drug treatment. It is unclear whether the aetiology of suicidal ideation lies in the mechanism of action of the antidepressant drug, a genetic susceptibility in the patient or even a mixture of both in a gene-by-drug interaction [Perroud et al., 2012]. Our results suggest that it is an inherent part of the genetic liability to depressive illness.

Since suicidal behavior is a spectrum of severity with heterogeneous genetic architecture, its decomposition by sex, age, number, or violence of attempts could lead to increased homogeneity, but much larger sample sizes would be required for these analyses. Furthermore, an integrative approach, incorporating genetic, epigenetic and environmental effects may be necessary to realistically capture the multifactorial nature of SA.

Molecular genetic studies on suicide attempt may contribute to the development of a biomarker risk model to be used alongside the clinical risk model of current practice in order to target intervention strategies to psychiatric patients at increased risk. Increased insight into the pathophysiology of suicidal behavior could also identify novel therapeutic targets.

In conclusion, the genetic architecture of SA and ideation is likely to be highly polygenic, with risk variants of small effect sizes which these GWAS studies were underpowered to detect, in a SNP-by-SNP analysis. Using polygenic scores as predictors in independent datasets is a more appropriate approach. Polygenic scores indicate that SA and ideation are unlikely to be different severities of the same spectrum. A genetic susceptibility to suicidal ideation could be part of the genetic aetiology of psychiatric disorders, while in contrast there is likely to be some independent genetic effects, which predispose to suicide attempt.
