# Supplementary figures and images for: Genetic relationships between suicide attempts, suicidal ideation and major psychiatric disorders: A genome-wide association and polygenic scoring study
Source: Am J Med Genet B Neuropsychiatr Genet. 2014 Jun 25;165(5):428–37. doi: 10.1002/ajmg.b.32247 (PMC4309466; doi:10.1002/ajmg.b.32247)

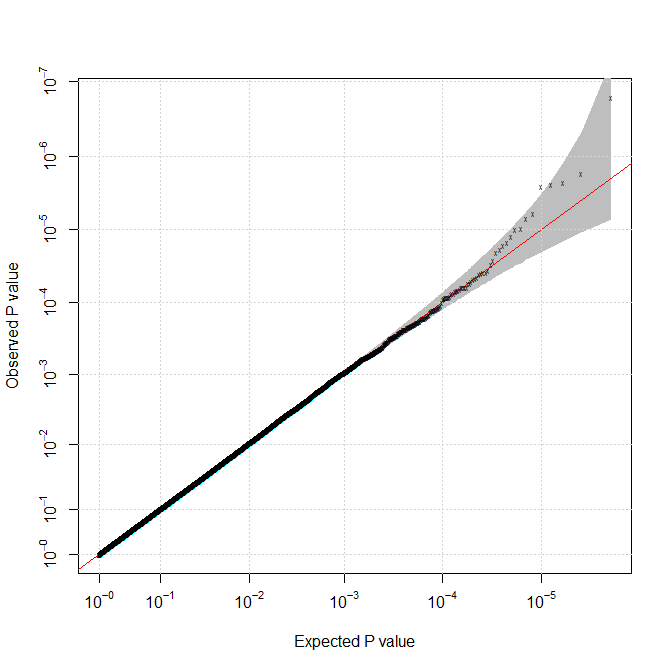

Supplement: Supplementary file 1 [file ajmg0165-0428-sd1.tif]

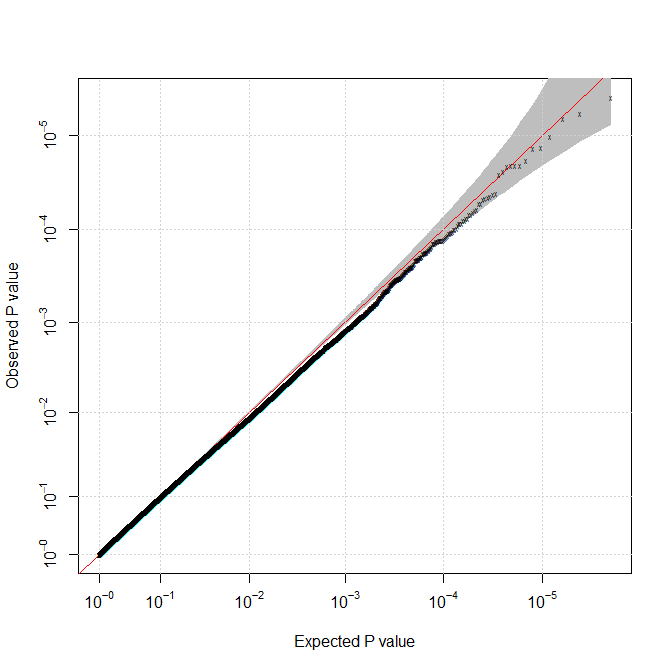

Supplement: Supplementary file 2 [file ajmg0165-0428-sd2.tif]

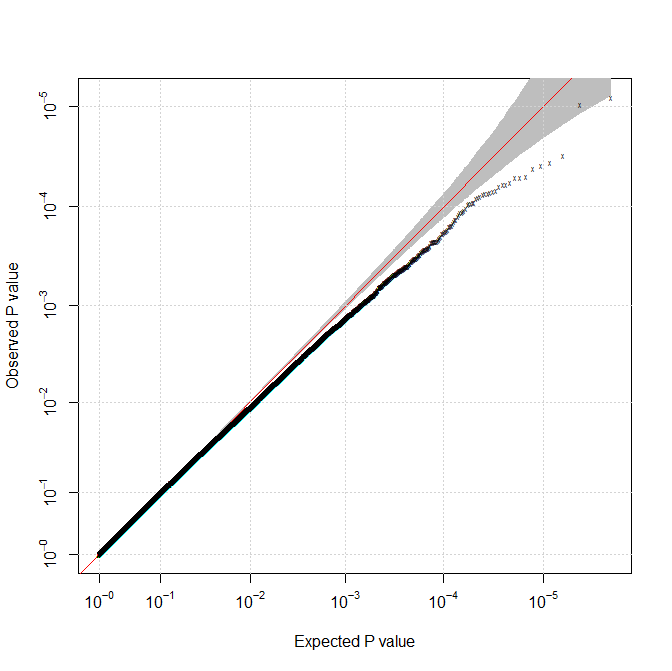

Supplement: Supplementary file 3 [file ajmg0165-0428-sd3.tif]

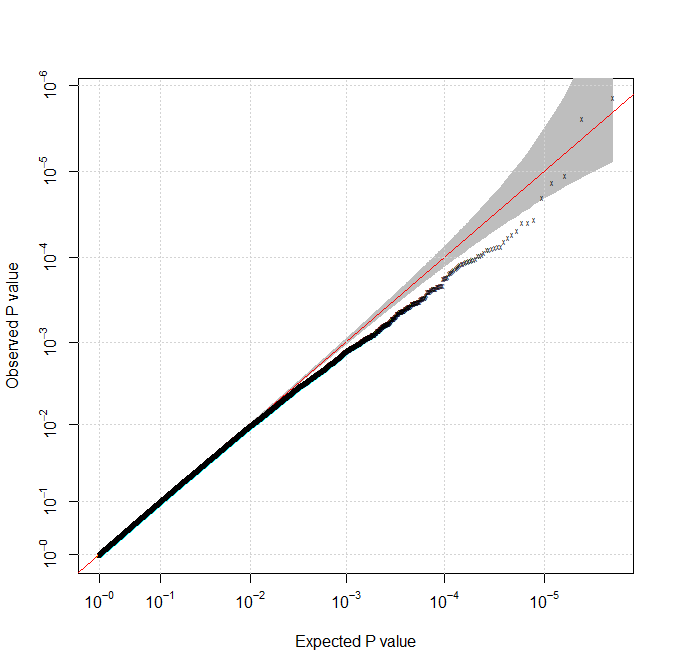

Supplement: Supplementary file 4 [file ajmg0165-0428-sd4.tif]

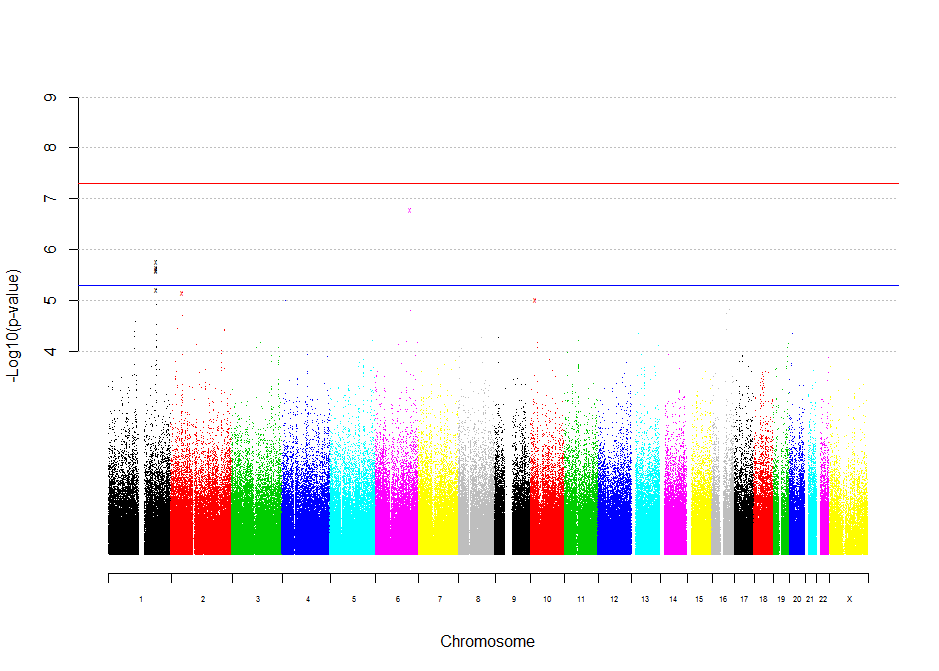

Supplement: Supplementary file 5 [file ajmg0165-0428-sd5.tif]

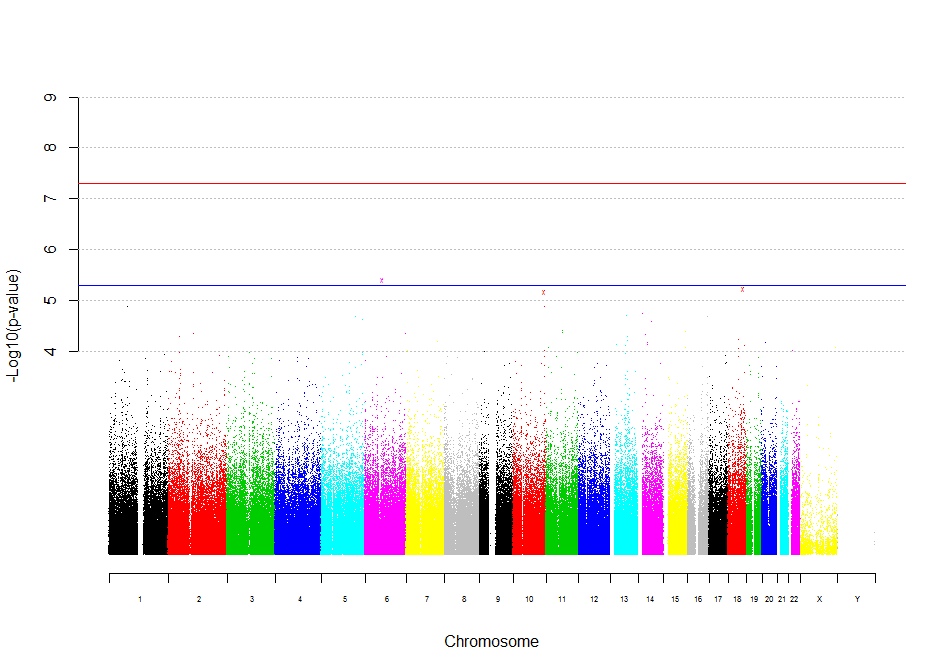

Supplement: Supplementary file 6 [file ajmg0165-0428-sd6.tif]

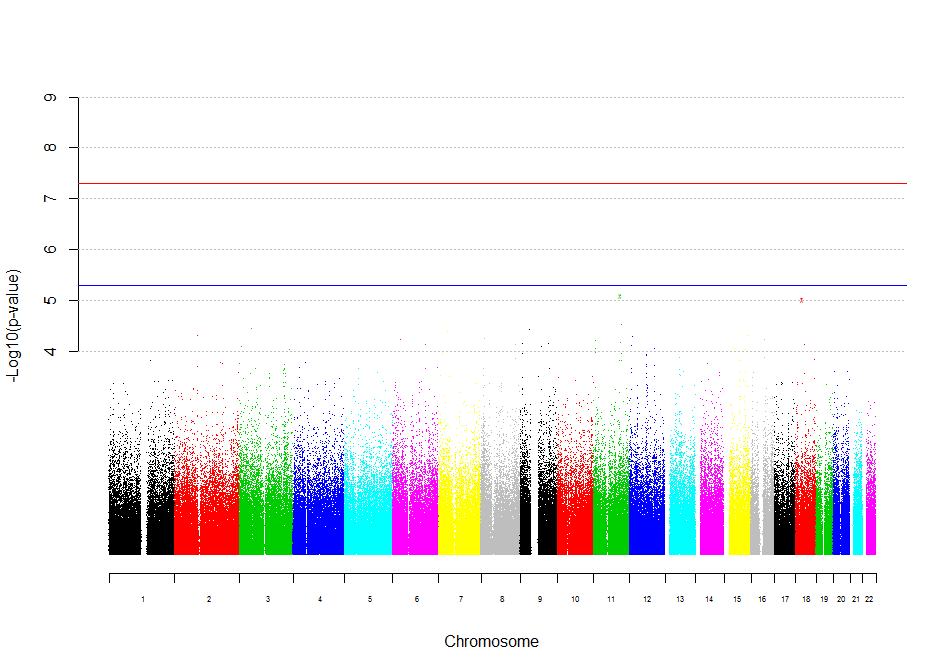

Supplement: Supplementary file 7 [file ajmg0165-0428-sd7.tif]

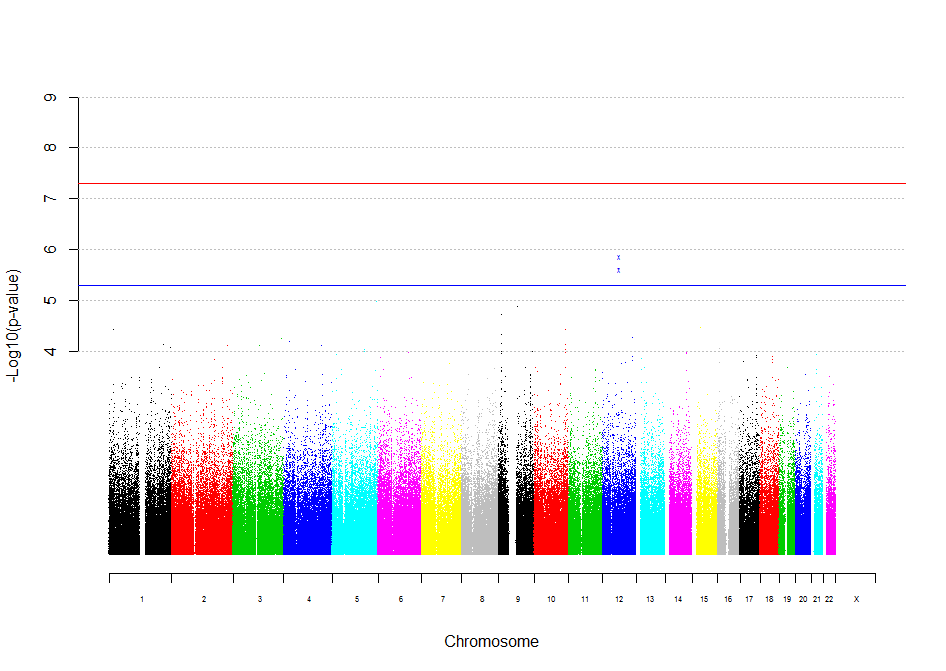

Supplement: Supplementary file 8 [file ajmg0165-0428-sd8.tif]

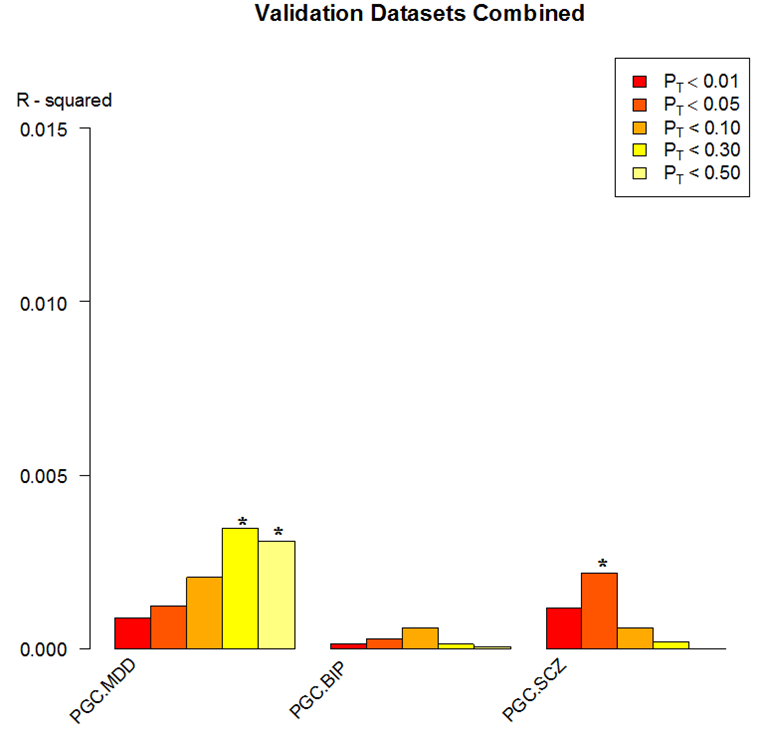

Supplement: Supplementary file 9 [file ajmg0165-0428-sd9.tif]
