# Supplementary material for: Genetic relationships between suicide attempts, suicidal ideation and major psychiatric disorders: A genome-wide association and polygenic scoring study
Source: Am J Med Genet B Neuropsychiatr Genet. 2014 Jun 25;165(5):428–37. doi: 10.1002/ajmg.b.32247 (PMC4309466; doi:10.1002/ajmg.b.32247)
Supplement: Supplementary file 10 [file ajmg0165-0428-sd10.docx]

| **Table SI: Selected genes and results of the candidate association study.** | | | |  |  |  |  |  |  |
| --- | --- | --- | --- | --- | --- | --- | --- | --- | --- |
| **Gene Symbol** | **Gene Name** | **Function** | **Rationale** | **References** | **No. SNPs tested** | **Most significant SNP** | **Tested Allele** | **P value** | **Odds ratio** |
| *SLC6A4* | Solute carrier family 6 member 4 | Serotonin transporter | Serotonin transporter functional promoter polymorphism (*HTTLPR*) associations with SA | Anguelova et al., 2003 | 535 | rs7210939 | C | 0.0001 | 1.32 |
| *NTRK2* | Neurotrophic tyrosine kinase receptor 2 | Cell differentiation | SNP associations with SA and suicidal ideation | Kohli et al., 2010 Perroud et al., 2012 | 35 | rs10868238 | T | 0.0185 | 0.83 |
| *TPH1* | Tryptophan hydroxylase 1 | Serotonin synthesis | SNP associations with SA | Bevillier et al., 2004 Clayden et al., 2012 Rujescu et al., 2003 | 1 | rs11024449 | T | 0.2064 | 0.90 |
| *TPH2* | Tryptophan hydroxylase 2 | Serotonin synthesis | CNS specific gene, increased expression in brains of depressed suicide victims | Bach-Mizrachi etal., 2008 | 15 | rs11615016 | G | 0.0308 | 0.73 |
| *HTR2A* | Serotonin receptor 2A | Serotonin signalling | SNP associations with impulsivity and SA | Salo et al., 2010 Saiz et al., 2010 | 10 | rs1928042 | G | 0.2770 | 1.11 |
| *ACP1* | Acid phosphatase 1 | Hydrolysis of protein tyrosine phosphatase | Locus association with SA | Willour et al., 2012 | 1 | rs12714402 | A | 0.8511 | 1.02 |
| *LRRTM4* | Leucine rich repeat transmembrane protein 4 | Nervous system development | Locus and SNP associations with SA | Hesselbrock et al., 2004 Zubenko et al., 2004 Willour et al., 2007 Willour et al., 2012 | 1 | rs6547148 | T | 0.7590 | 0.98 |
| *COMT* | Catechol-O-methyltransferase | Catecholamine metabolism | SNP associations with SA, irritablity and impulsivity | Kia-Keating et al., 2007 Calati et al., 2011 Salo et al., 2010 | 1 | rs737866 | C | 0.3812 | 0.93 |
| *GABRG2* | Gamma-aminobutyric acid (GABA) A receptor, gamma 2 | Inhibitory neurotransmission | Increased expression in brains of depressed suicide victims | Sequeira et al., 2009 Choudery et al., 2005 Merali et al., 2004 | 6 | rs211029 | C | 0.1285 | 0.89 |
| *CRHR1* | Corticotropin-releasing hormone receptor 1 | Stress response | Decreased expression in suicide brain | Merali et al., 2004 | 2 | rs242942 | T | 0.0955 | 1.21 |
| *GRIA3* | Glutamate Receptor Ionotropic AMPA3 | Excitatory neurotransmission | SNP associations with treatment emergent suicidal ideation | Laje et al., 2007 Menke et al., 2008 | 3 | rs5911622 | T | 0.0407 | 0.84 |
| *BDNF* | Brain-derived neurotrophic factor | Neuronal growth and plasticity | SNP associations with SA and suicidal ideation | Kim et al., 2008 Sarchiapone et al., 2008 Zai et al., 2012 Perroud et al., 2009 | 1 | rs6265 | T | 0.3574 | 1.09 |
| *NGFR* | Nerve growth factor receptor | Neuronal survival and death | SNP associations with SA | Kenugi et al., 2004 Perlis et al., 2010 | 2 | rs741071 | T | 0.4715 | 1.06 |
| *ABCG1* | ATP binding cassette subfamily G member 1 | Lipid transport | SNP associations with anger and aggression | Gietl et al., 2007 | 19 | rs532345 | T | 0.0110 | 1.21 |
| *GRIK2* | Glutamate Receptor Ionotropic Kainate 2 | Excitatory neurotransmission | SNP associations with treatment emergent suicidal ideation | Laje et al., 2007 Menke et al., 2008 | 32 | rs2518251 | G | 0.0215 | 1.21 |
| *SLC1A2* | Excitatory Amino Acid Transporter 1 A2 | Glutamate transporter | SNP association with SA | Murphy et al., 2011 | 22 | rs2273689 | A | 0.0010 | 0.77 |
| *DNMT3B* | DNA methyltransferase 3B | De novo DNA methylation | SNP association with SA, increased expression in brains of suicide victims | Murphy et al., 2012 Poulter et al., 2008 | 1 | rs2424908 | T | 0.0772 | 0.84 |
| *ABI3BP* | Abl-interactor family member 3 binding protein | Possible role in apoptosis and senescence | SNP associations with SA | Perlis et al., 2010 | 35 | rs1847393 | A | 0.0152 | 1.20 |
| *SLC4A4* | Solute carrier family 4, sodium bicarbonate co-transporter, member 4 | Electrolyte homeostasis | SNP associations with SA | Perlis et al., 2010 | 13 | rs1377287 | G | 0.0103 | 0.71 |
| SA-suicide attempt | |  |  |  |  |  |  |  |  |
