# Supplementary material for: Genetic relationships between suicide attempts, suicidal ideation and major psychiatric disorders: A genome-wide association and polygenic scoring study
Source: Am J Med Genet B Neuropsychiatr Genet. 2014 Jun 25;165(5):428–37. doi: 10.1002/ajmg.b.32247 (PMC4309466; doi:10.1002/ajmg.b.32247)
Supplement: Supplementary file 11 [file ajmg0165-0428-sd11.docx]

| **Table SII: Sample characteristics and percentage power to detect associations at genome-wide and suggestive significance.** | | | | |
| --- | --- | --- | --- | --- |
| **Sample** | **No. Cases** | **No. Controls** | **P< 5x10^-8^*** | **P< 5x10^-6^*** |
| RADIANT | 251 | 1772 | 14% | 42% |
| GSK-Munich | 106 | 701 | 0% | 4% |
| BACCs | 69 | 371 | 0% | 1% |
| Meta-analysis^^^ | 426 | 2844 | 59% | 87% |
| GENDEP | 237 | 510 | 25% | 59% |
| *P< 5x10^-8^= genome-wide significance. P< 5x10^-6^= suggestive significance. ^^^ RADIANT, GSK-Munich and BACCs studies. | | | | |
