# Supplementary material for: Genetic relationships between suicide attempts, suicidal ideation and major psychiatric disorders: A genome-wide association and polygenic scoring study
Source: Am J Med Genet B Neuropsychiatr Genet. 2014 Jun 25;165(5):428–37. doi: 10.1002/ajmg.b.32247 (PMC4309466; doi:10.1002/ajmg.b.32247)
Supplement: Supplementary file 12 [file ajmg0165-0428-sd12.docx]

| **Table SIII: SNPs most associated with suicide attempt or ideation in an additive genetic model showing only the top SNP from each** | | | | | | |
| --- | --- | --- | --- | --- | --- | --- |
| **genomic region.** | | | | |  |  |
| **CHR** | **SNP** | **BP** | **Tested allele** | **Allele freq*** | **OR (CI)** | **P value** |
| **RADIANT** |  |  |  |  |  |  |
| **6** | **rs203136** | **138647945** | **G** | **0.365** | **1.66 (1.37-2.01)** | **1.60E-07** |
| **1** | **rs12751302** | **189813197** | **T** | **0.341** | **0.58 (0.46-0.72)** | **1.71E-06** |
| 2 | rs4953249 | 45846361 | G | 0.101 | 1.86 (1.40-2.45) | 6.99E-06 |
| 10 | rs703088 | 21180261 | A | 0.051 | 2.17 (1.54-3.06) | 9.64E-06 |
| 4 | rs17387100 | 15604223 | G | 0.078 | 1.92 (1.40-2.56) | 1.00E-05 |
| 16 | rs16972539 | 72362202 | T | 0.136 | 1.70 (1.34-2.17) | 1.52E-05 |
| 16 | rs8061077 | 57255287 | T | 0.211 | 1.58 (1.28-1.96) | 1.87E-05 |
| 1 | rs6583045 | 108088494 | A | 0.410 | 1.50 (1.24-1.81) | 2.65E-05 |
| 2 | rs2305450 | 29236878 | G | 0.090 | 1.80 (1.36-2.39) | 3.61E-05 |
| 16 | rs7191331 | 57271299 | A | 0.193 | 1.57 (1.27-1.95) | 3.81E-05 |
| **GSK-Munich** |  |  |  |  |  |  |
| **6** | **rs9351947** | **73582070** | **T** | **0.253** | **2.04 (1.51-2.77)** | **3.89E-06** |
| 18 | rs683277 | 64099632 | G | 0.061 | 2.91 (1.83-4.62) | 5.77E-06 |
| 10 | rs10829429 | 130256204 | C | 0.354 | 1.93 (1.45-2.58) | 6.58E-06 |
| 16 | rs2017161 | 84470594 | C | 0.298 | 1.96 (1.45-2.65) | 1.03E-05 |
| 1 | rs6696780 | 76443928 | A | 0.153 | 2.12 (1.51-2.97) | 1.33E-05 |
| 14 | rs10140287 | 21139619 | C | 0.447 | 1.86 (1.40-2.48) | 1.83E-05 |
| 13 | rs1323910 | 66410878 | T | 0.196 | 2.08 (1.48-2.93) | 2.05E-05 |
| 5 | rs10070905 | 143020165 | A | 0.236 | 1.95 (1.43-2.66) | 2.10E-05 |
| 5 | rs33376 | 171034331 | A | 0.444 | 1.88 (1.40-2.53) | 2.38E-05 |
| 14 | rs192849 | 58210618 | G | 0.376 | 0.49 (0.35-0.68) | 2.60E-05 |
| **BACCs** |  |  |  |  |  |  |
| 11 | rs1218927 | 100699657 | G | 0.066 | 4.17 (2.22-7.81) | 8.07E-06 |
| 18 | rs11661081 | 22684030 | A | 0.093 | 3.35 (1.96-5.74) | 9.55E-06 |
| 11 | rs1939900 | 106977193 | T | 0.083 | 3.24 (1.86-5.64) | 3.06E-05 |
| 3 | rs7648681 | 45652402 | A | 0.394 | 0.40 (0.26-0.62) | 3.56E-05 |
| 9 | rs366887 | 36532368 | C | 0.253 | 2.27 (1.53-3.35) | 3.89E-05 |
| 7 | rs13247717 | 34294279 | G | 0.292 | 0.35 (0.21-0.58) | 4.13E-05 |
| 15 | rs4887403 | 86626702 | T | 0.142 | 2.58 (1.63-4.09) | 5.00E-05 |
| 2 | rs11675205 | 85381939 | A | 0.280 | 2.23 (1.51-3.29) | 5.09E-05 |
| 12 | rs4763291 | 12644945 | C | 0.064 | 3.30 (1.85-5.88) | 5.12E-05 |
| 8 | rs1841907 | 13982707 | T | 0.090 | 3.39 (1.87-6.14) | 5.69E-05 |
| **GENDEP** |  |  |  |  |  |  |
| **12** | **rs10748045** | **65102406** | **G** | **0.354** | **1.78 (1.41-2.25)** | **1.37E-06** |
| 5 | rs478253 | 176238284 | A | 0.388 | 1.73 (1.35-2.22) | 1.09E-05 |
| 9 | rs11143230 | 74077523 | C | 0.353 | 1.69 (1.33-2.14) | 1.34E-05 |
| 9 | rs10960522 | 12192003 | C | 0.393 | 1.67 (1.32-2.11) | 1.97E-05 |
| 15 | rs11854649 | 33268546 | A | 0.063 | 2.52 (1.62-3.92) | 3.55E-05 |
| 1 | rs10916754 | 20496650 | A | 0.183 | 1.81 (1.36-2.40) | 3.84E-05 |
| 10 | rs11200059 | 123405580 | A | 0.393 | 1.61 (1.28-2.02) | 3.86E-05 |
| 12 | rs2555283 | 118166865 | C | 0.472 | 0.61 (0.48-0.78) | 5.38E-05 |
| 3 | rs13340153 | 191553513 | A | 0.060 | 0.25 (0.13-0.49) | 5.79E-05 |
| 4 | rs13112011 | 25182217 | C | 0.075 | 2.30 (1.53-3.48) | 6.42E-05 |
| CHR-chromosome, BP-base pair, OR-odds ratio, CI-confidence interval *Allele frequencies are from each individual study. | | | | | | |
