# Supplementary material for: Genetic relationships between suicide attempts, suicidal ideation and major psychiatric disorders: A genome-wide association and polygenic scoring study
Source: Am J Med Genet B Neuropsychiatr Genet. 2014 Jun 25;165(5):428–37. doi: 10.1002/ajmg.b.32247 (PMC4309466; doi:10.1002/ajmg.b.32247)
Supplement: Supplementary file 13 [file ajmg0165-0428-sd13.docx]

**Supplementary Material**

**Genetic Relationships between Suicide Attempts, Suicidal Ideation and Major Psychiatric Disorders: a Genome-wide Association and Polygenic Scoring Study**

**Authors**

Niamh Mullins^1^**^*^**, Nader Perroud^2^, Rudolf Uher^1,3^, Amy W. Butler^1,4^, Sarah Cohen-Woods^5^, Margarita Rivera^1,6^, Karim Malki^1^, Jack Euesden^1^, Robert A. Power^1^, Katherine E. Tansey^7^, Lisa Jones^8^, Ian Jones^7^, Nick Craddock^7^, Michael J. Owen^7^, Ania Korszun^9^, Michael Gill^10^, Ole Mors^11^, Martin Preisig^12^, Wolfgang Maier^13^, Marcella Rietschel^13,14^, John P. Rice^15^, Bertram Müller-Myhsok^16^, Elisabeth B. Binder^16^, Susanne Lucae^16^, Marcus Ising^16^, Ian W. Craig^1^, Anne E. Farmer^1^, Peter McGuffin^1^, Gerome Breen^1,17^, Cathryn M. Lewis^1,18^

**Corresponding Author:** Niamh Mullins, MRC Social, Genetic and Developmental Psychiatry Centre, Institute of Psychiatry, King's College London, 16 De Crespigny Park, London SE5 8AF, United Kingdom. Email: [Niamh.mullins@kcl.ac.uk](mailto:Niamh.mullins@kcl.ac.uk) Phone: 0044 20 7848 5121

Quality Control

The genomic control λ values were all close to 1, indicating adequate control for population stratification (Figures S1-S4).

Candidate association study

Table SI shows the 19 genes selected from the literature for a candidate association study, along with their function, rationale for selection and references. Most genes selected are the classical candidates involved in neurotransmitter systems and have also been associated with suicide attempt or suicidal ideation. Some are novel associations from loci implicated by linkage studies or GWAS on suicide, though not in any of the datasets used for this analysis.

The candidate gene investigation was carried out in the meta-analysis of the GWAS results in the RADIANT, GSK-Munich and BACCs samples using 735 SNPs in approximate linkage equilibrium in the 19 selected genes. No SNP reached the Bonferroni corrected significance threshold of 6.80 x 10^-5^. Table S1 shows the most significant SNP tested in each gene along with its P value and odds ratio.

Power Calculations

After QC procedures, the RADIANT GWAS included 503,016 SNPs and GSK-Munich included 484,091 SNPs. The BACCs and GENDEP studies tested 476,511 and 494,742 SNPs respectively.

The power of each study and the meta-analysis to detect associations at both genome-wide (P < 5x10^-8^) and suggestive significance (P < 5x10^-6^), for a minor allele with a frequency of 0.2 and an effect size of 1.5 was calculated (Table SII). Individual studies were all underpowered to detect these associations. The meta-analysis had sufficient power to detect associations at the suggestive significance level (87%).

Genome-Wide Association Studies

A genome-wide association study was carried out in each sample under an additive genetic model. Table SIII shows the ten SNPs most strongly associated with suicide attempt or ideation from each genomic region. Results of the RADIANT and GENDEP analyses have previously been published but are shown here for comparison [[23](#_ENREF_23), [24](#_ENREF_24)]. No SNP reached significance at the genome-wide level in any of the four studies. rs9351947 on chromosome 6 was the only SNP reaching suggestive significance in the GSK-Munich sample (P = 3.89 x 10^-6^; OR = 2.04; C.I. 1.51 – 2.77) (Table SIII). No SNPs in the BACCs study reached the suggestive significance level (Table SIII).

Manhattan plots were generated to graphically view the results of the additive GWAS in each sample (Figures S5-8). The blue line indicates the level of suggestive significance (P = 5x10^-6^) and the red line indicates genome-wide significance (P = 5x10^-8^).

Polygenic Score Analysis

The RADIANT, GSK-Munich and BACCs validation datasets were merged into a single dataset in order to increase the precision of prediction. This sample has the same characteristics as the meta-analysis (n=3270) and includes 426 SA cases. Scores for MDD, BIP and SCZ from the PGC were tested as predictors of SA in this combined validation dataset (Figure S9). PGC-MDD scores showed significant predictive ability for SA, the largest R^2^ being 0.3% at P_T_ < 0.3 (P = 0.013). Scores from PGC-SCZ also showed significant prediction (R^2^ = 0.2% at P_T_ < 0.05, P = 0.05), but the score was lower in SA cases than in non-SA cases, consistent with the previous analysis in the GENDEP sample.

Bibliography

1. Kohli, M.A., et al., *Association of genetic variants in the neurotrophic receptor-encoding gene NTRK2 and a lifetime history of suicide attempts in depressed patients.* Arch Gen Psychiatry, 2010. **67**(4): p. 348-59.

2. Clayden, R.C., et al., *The association of attempted suicide with genetic variants in the SLC6A4 and TPH genes depends on the definition of suicidal behavior: a systematic review and meta-analysis.* Transl Psychiatry, 2012. **2**: p. e166.

3. Bach-Mizrachi, H., et al., *Elevated expression of tryptophan hydroxylase-2 mRNA at the neuronal level in the dorsal and median raphe nuclei of depressed suicides.* Mol Psychiatry, 2008. **13**(5): p. 507-13, 465.

4. Salo, J., et al., *The interaction between serotonin receptor 2A and catechol-O-methyltransferase gene polymorphisms is associated with the novelty-seeking subscale impulsiveness.* Psychiatr Genet, 2010. **20**(6): p. 273-81.

5. Saiz, P.A., et al., *Association between the A-1438G polymorphism of the serotonin 2A receptor gene and nonimpulsive suicide attempts.* Psychiatr Genet, 2008. **18**(5): p. 213-8.

6. New, A.S., et al., *Suicide, impulsive aggression, and HTR1B genotype.* Biol Psychiatry, 2001. **50**(1): p. 62-5.

7. Ramboz, S., et al., *5-HT1B receptor knock out--behavioral consequences.* Behav Brain Res, 1996. **73**(1-2): p. 305-12.

8. Bouwknecht, J.A., et al., *Absence of 5-HT(1B) receptors is associated with impaired impulse control in male 5-HT(1B) knockout mice.* Biol Psychiatry, 2001. **49**(7): p. 557-68.

9. Kia-Keating, B.M., S.J. Glatt, and M.T. Tsuang, *Meta-analyses suggest association between COMT, but not HTR1B, alleles, and suicidal behavior.* Am J Med Genet B Neuropsychiatr Genet, 2007. **144B**(8): p. 1048-53.

10. Calati, R., et al., *Catechol-o-methyltransferase gene modulation on suicidal behavior and personality traits: review, meta-analysis and association study.* J Psychiatr Res, 2011. **45**(3): p. 309-21.

11. Sequeira, A., et al., *Global brain gene expression analysis links glutamatergic and GABAergic alterations to suicide and major depression.* PLoS One, 2009. **4**(8): p. e6585.

12. Choudary, P.V., et al., *Altered cortical glutamatergic and GABAergic signal transmission with glial involvement in depression.* Proc Natl Acad Sci U S A, 2005. **102**(43): p. 15653-8.

13. Merali, Z., et al., *Dysregulation in the suicide brain: mRNA expression of corticotropin-releasing hormone receptors and GABA(A) receptor subunits in frontal cortical brain region.* J Neurosci, 2004. **24**(6): p. 1478-85.

14. Laje, G., et al., *Genetic markers of suicidal ideation emerging during citalopram treatment of major depression.* Am J Psychiatry, 2007. **164**(10): p. 1530-8.

15. Menke, A., et al., *Genetic markers within glutamate receptors associated with antidepressant treatment-emergent suicidal ideation.* Am J Psychiatry, 2008. **165**(7): p. 917-8.

16. Wasserman, D., et al., *The serotonin 1A receptor C(-1019)G polymorphism in relation to suicide attempt.* Behav Brain Funct, 2006. **2**: p. 14.

17. Kim, B., et al., *Brain-derived neurotrophic factor Val/Met polymorphism and bipolar disorder. Association of the Met allele with suicidal behavior of bipolar patients.* Neuropsychobiology, 2008. **58**(2): p. 97-103.

18. Sarchiapone, M., et al., *Association of polymorphism (Val66Met) of brain-derived neurotrophic factor with suicide attempts in depressed patients.* Neuropsychobiology, 2008. **57**(3): p. 139-45.

19. Zai, C.C., et al., *The brain-derived neurotrophic factor gene in suicidal behaviour: a meta-analysis.* Int J Neuropsychopharmacol, 2012. **15**(8): p. 1037-42.

20. Perroud, N., et al., *Suicidal ideation during treatment of depression with escitalopram and nortriptyline in genome-based therapeutic drugs for depression (GENDEP): a clinical trial.* BMC Med, 2009. **7**: p. 60.

21. Gietl, A., et al., *ABCG1 gene variants in suicidal behavior and aggression-related traits.* Eur Neuropsychopharmacol, 2007. **17**(6-7): p. 410-6.

22. Murphy, T.M., et al., *Genetic variation in DNMT3B and increased global DNA methylation is associated with suicide attempts in psychiatric patients.* Genes Brain Behav, 2012.

23. Perroud, N., et al., *Genome-wide association study of increasing suicidal ideation during antidepressant treatment in the GENDEP project.* Pharmacogenomics J, 2012. **12**(1): p. 68-77.

24. Schosser, A., et al., *Genomewide association scan of suicidal thoughts and behaviour in major depression.* PLoS One, 2011. **6**(7): p. e20690.
